# Joint Action Syntax in Japanese Martial Arts

**DOI:** 10.1371/journal.pone.0072436

**Published:** 2013-09-04

**Authors:** Yuji Yamamoto, Keiko Yokoyama, Motoki Okumura, Akifumi Kijima, Koji Kadota, Kazutoshi Gohara

**Affiliations:** 1 Research Center of Health, Physical Fitness, and Sports, Nagoya University, Nagoya, Japan; 2 Division of Applied Physics, Department of Engineering, Hokkaido University, Sapporo, Japan; 3 Research Fellowship Division, Japan Society for the Promotion of Science, Tokyo, Japan; 4 Faculty of Education, Shizuoka University, Shizuoka, Japan; 5 Graduate School of Education, University of Yamanashi, Kofu, Japan; 6 Graduate School of Medicine, Osaka University, Toyonaka, Japan; Hungarian Academy of Sciences, Hungary

## Abstract

Participation in interpersonal competitions, such as fencing or Japanese martial arts, requires players to make instantaneous decisions and execute appropriate motor behaviors in response to various situations. Such actions can be understood as complex phenomena emerging from simple principles. We examined the intentional switching dynamics associated with continuous movement during interpersonal competition in terms of their emergence from a simple syntax. Linear functions on return maps identified two attractors as well as the transitions between them. The effects of skill differences were evident in the second- and third-order state-transition diagrams for these two attractors. Our results suggest that abrupt switching between attractors is related to the diverse continuous movements resulting from quick responses to sudden changes in the environment. This abrupt-switching-quick-response behavior is characterized by a joint action syntax. The resulting hybrid dynamical system is composed of a higher module with discrete dynamics and a lower module with continuous dynamics. Our results suggest that intelligent human behavior and robust autonomy in real-life scenarios are based on this hybrid dynamical system, which connects interpersonal coordination and competition.

## Introduction

Nonlinear dynamics has revealed that complex phenomena, from chemical reactions to the neural networks in the brain, emerge from simple principles. Examples of complexity theory include self-organization in thermodynamics theory [1], the slaving-principle in lasers [2], spatiotemporal chaos in fluid dynamics [3], and the synchronization of nonlinear-coupled oscillators [4]–[6]. Humans are considered to be complex systems as well. In response to various situations, people make instantaneous decisions and execute appropriate motor behaviors. Typical examples of this include the processes involved in interpersonal competition, such as fencing or Japanese martial arts originating from Samurai traditions.

The ability of humans to control complex cognitive processes, which is essential for what we recognize as intelligent behavior (e.g., decision making), depends on the prefrontal cortex (PFC) [7]–[9]. All goal-directed behaviors are learned and thus depend on a cognitive system that can grasp the rules of a game, the goals available, and the means used to achieve these goals. To this end, PFC activity exerts a top-down influence by providing excitation signals to bias other brain systems towards task-relevant information. This suggests that the PFC plays a role in the mapping of sensory inputs and internal states, such as the mapping between the current motivational state and memories or voluntary actions. PFC mapping can be described by the Hidden Markov Model (HMM), which holds that switches between states proceed according to conditional probabilities [10]. The HMM has been widely used to construct probabilistic language models in natural language processing and computational linguistics [11]; this model is regarded as an automation model in complex sciences. Human intentional dynamics and decision making have been modeled by neuropercolation based on the graph theory [12], which is a generalization of cellular automata [13], [14] (i.e., PFC activity is considered to be a discrete dynamical system).

In contrast, complex human movements have been examined using the continuous model of a dynamical system. The Haken-Kelso-Bunz (HKB) model [15] was derived from the theory of nonlinear oscillators and synergetics [2], [16], [17], which was based on the observation of phase transitions for two-finger experiments [18], [19]. These experiments have shown the abrupt change from one stable state to another for critical values as the movement frequency gradually increases. In a discrete dynamical system, this frequency would be a bifurcation parameter, and the phase differences of movements would be regarded as collective variables. The HKB model is based on the synchronization of nonlinear-coupled oscillators. If the system consists of two coupled oscillators, then the system has two stable states: in-phase synchronization and anti-phase synchronization. Pitchfork bifurcation describes the change from these two stable states to one stable state (i.e., from anti- and in-phase synchronization to in-phase synchronization). This model is commonly applied to interlimb coordination and/or perception-action coordination in human movements [20]. Interpersonal coordination has also been studied in terms of the coupling between two oscillators using visual information based on this model [21]–[23]. For example, synchronization among six people during the swinging of rocking chairs was examined using the Kuramoto order parameter [24], [25]. Synchronized patterns among three people who communicated via perceptual information during sports activities was also confirmed based on the coupled-oscillators approach derived from symmetric Hopf bifurcation group theory [26], [27].

However, little is known about the dynamics underlying the continuous abrupt switching behavior observed in martial arts in which both quick decision making and execution are required. In this study, to clarify the intentional switching dynamics during interpersonal competition we observed a time series of the interpersonal distances (IPDs) between two players based on their moving trajectories during 24 matches of Japanese fencing or kendo from the viewpoint of a hybrid dynamical system [28], [29]. Analogous to words and sentences in language, numerous complex behavioral patterns during interpersonal competition could be organized by syntactical rules that can be considered “action syntax” [30], [31]. Grooming in rodents has been examined as “action syntax” and regarded as a Markov chain [32]–[34]; however, these stereotypical actions can be generated by a relatively simple feed-forward excitatory mechanism that cannot adapt to environmental changes, such as interpersonal competition. In response to various situations, very large numbers of movements can be generated in large strongly recurrent connected systems equipped with appropriate rules [31]. We first attempted to extend stereotypical “action syntax” to adaptive “joint action syntax” during complex interpersonal competition characterized by quick decision making and rapidly executed actions.

## Methods

### Participants

Twelve male members of the University of Tsukuba kendo club participated in the experiment. This club has won the kendo championship in the annual team competition for all Japanese universities three times since 2000. All participants were healthy. Six regular players on the team had expert status; their average age was 20.67±0.75 years, and they had an average of 14.17±1.77 years of kendo experience. Six substitute players held intermediate status; their average age was 21.17±1.57 years, and they had an average of 13.83±0.69 years of kendo experience. All participants provided written informed consent prior to the experiments. The participants in this study have given written informed consent for their photographs to be published, as outlined in the PLOS consent form. Procedures were approved by the Internal Review Board at the Research Center of Health, Fitness, and Sports at Nagoya University and conformed to the principles expressed in the Declaration of Helsinki.

### Task

**Figure 1:**
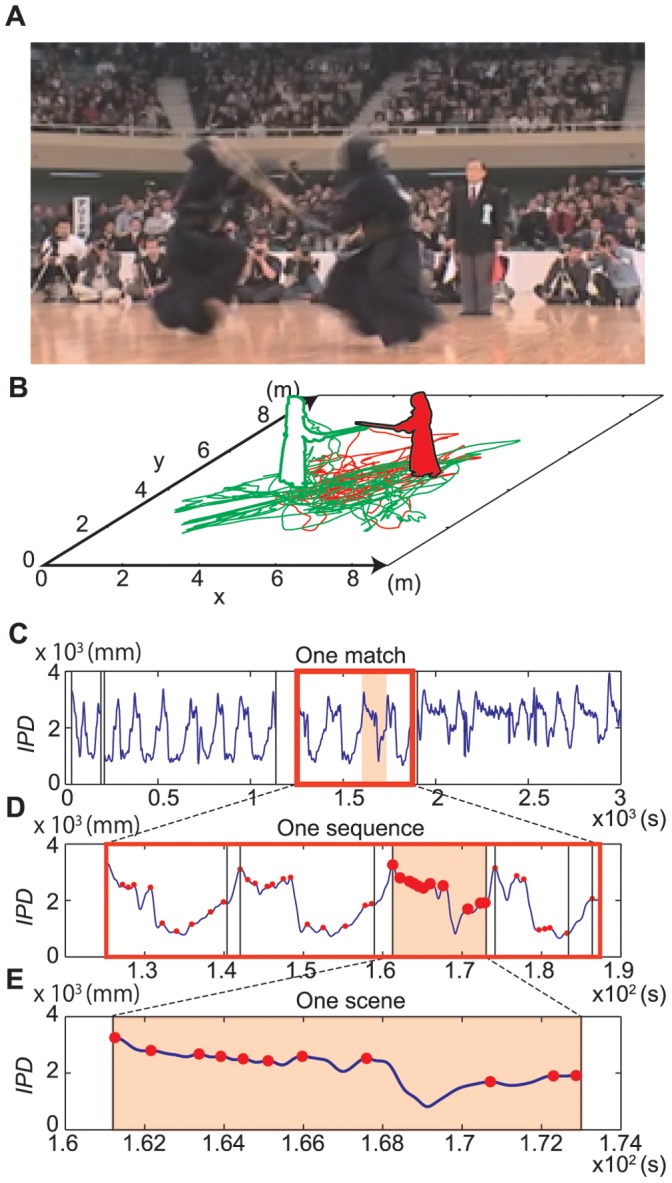
Scene selection. (**A**) Kendo match. (**B**) Trajectories of two players during a kendo match over a 5-min period in a two-dimensional plane (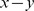). (**C**) Time series of interpersonal distance (IPD) for one match. (**D**) Time series of IPD for one sequence eliminating unrelated scenes. (**E**) Time series of IPD for one scene that begins with the contestants at the greatest distance from one another and ends with them coming together in a striking action.

Each of the six players at both skill levels were matched against four different opponents of the same skill level. If one player had been matched against five other players in a round-robin system, a total of 15 matches would have been played. However, because no player would compete against one particular player, a total of 12 matches were played at each level. Following official kendo rules, each match lasted 5 min and was played on a square court with 11.00-m sides (Figure 1A–B, Figure S1A, and Video S1). Each match was judged by three referees. We observed 37.42±5.45 instances of striking and 2.33±1.25 points per match for experts and 37.42±10.11 instances of striking and 2.17±1.67 points per match for intermediate players.

### Experimental Devices

Players’ movement trajectories were recorded using an optical motion capture system with eight different cameras (100 Hz, OQUS300, Qualysis, Inc.) and a Movie camera positioned at various locations near the court. Large reflective markers were attached to the back of each player’s head, the back of his waist, both ankles, the right knee, and the top of the shinai or fencing foil to detect movement (Figure S1B, S1C, and Video S1).

### Scene Selection

First, an experimenter eliminated unrelated scenes in which the match was stopped by the referees. Additionally, scenes in which the reflective markers could not be seen because the players were outside the camera angles were also removed (Figure 1C). As a result, the analyzed data averaged 4 min 19 s±11 s for experts and 4 min 26 s±22 s for intermediate players per match, although the matches lasted 5 min from start to finish. Each of the 12 matches was divided into 67 and 54 sequences for expert and intermediate competitors, respectively (Figure 1D). We divided each scene (which included only one striking action) because interpersonal competition in kendo is interrupted by the striking action, and movements after the striking action were considered transitions to the next competition. We detected positive and negative peaks in each sequence to identify the striking action. The positive peaks corresponded to moments of approaching movements between competitors; negative peaks corresponded to moments of detaching movements. A quick detaching movement was defined as a movement in which two adjacent peaks of IPD time series had spread more than 1 m within 1,500 ms; these movements were eliminated. As a result, each scene started with the farthest interpersonal distance and ended with the nearest distance for striking or with the middle distance for slow detachment (Figure 1E). Scenes that included fewer than four positive peaks were excluded from further analysis. As a result, 184 scenes involving experts and 162 scenes involving intermediate players remained. The longest scene was 38.2 and 31.8 s for experts and intermediate players, respectively. The shortest scene was 4.1 and 4.2 s for experts and intermediate players, respectively.

### State Variables

The trajectory of the player’s head position was expressed as time-dependent vectors 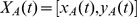 for player A and 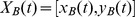 for player B. These time-series vectors were calculated using software (Qualysis Track Manager, Qualysis, Inc.) and flattened using a fourth-order Butterworth filter with a cutoff frequency of 6 Hz. The time series for the Euclidean distance 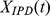 between two players was calculated using the following equation:
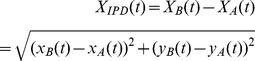
(1)where 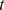 is a series of 0.01-s sampling intervals.

Displacement and velocity are state variables that represent the behavior of the system. 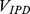, that is, change in 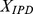, was calculated using the following general equation:
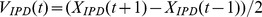
(2)


However, a relatively large variance is required because 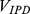 is calculated at the peaks of 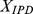 in the return map analysis. Additionally, 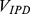 was independent of 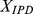 to create one state variable.

To determine the delay from 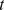, 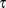, 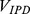 was calculated using the following equation:
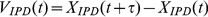
(3)


The 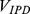 was calculated for 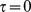 to 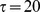. The variance of 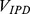 and the correlation coefficient between 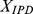 in each 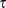 were calculated. Figure S2 shows the results. The first crossing point occurred at 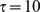 and 0.1 s. The 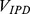 corresponding to this 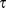 had a relatively large variance and was independent in minimum delay from 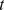.

**Figure 2:**
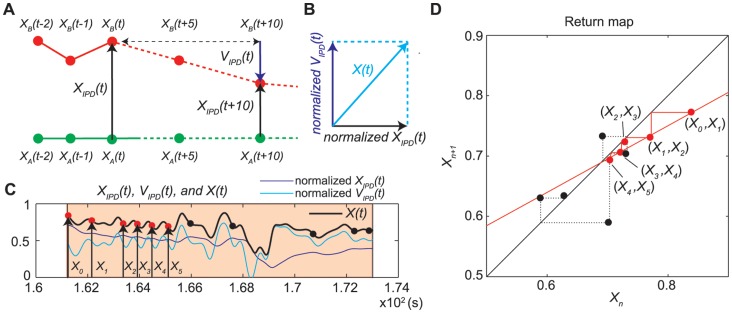
Schematic representation of state variables (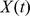). (**A**) Schematic representation of 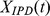 and 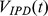. (**B**) Schematic representation of the state variables. (**C**) Blue, cyan, and black lines show a time series of normalized 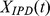, normalized 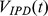, and 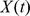, respectively, for a 12-s scene that had more than five peaks. The red and black circles show the corresponding values of 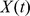 to the peaks of 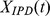. (**D**) Return map of the time series of the observed data, 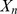 versus 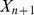 using the amplitude of 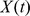 at the peaks of 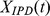 corresponding to the series of points (red and black circles) in panel shown in **C**.


As a result, 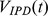 was calculated using the following equation;
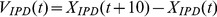
(4)



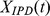
 and 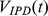 were calculated for the entire duration of each of the 24 matches (Figure 2A). Both 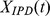 and 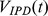 were normalized between 0 and 1, and state variables 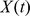 were calculated as composite vectors of two time-dependent vectors using the following equation (Figure 2B):

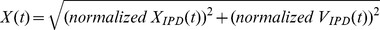
(5)

### Return Map and State Transition Analysis

**Figure 3:**
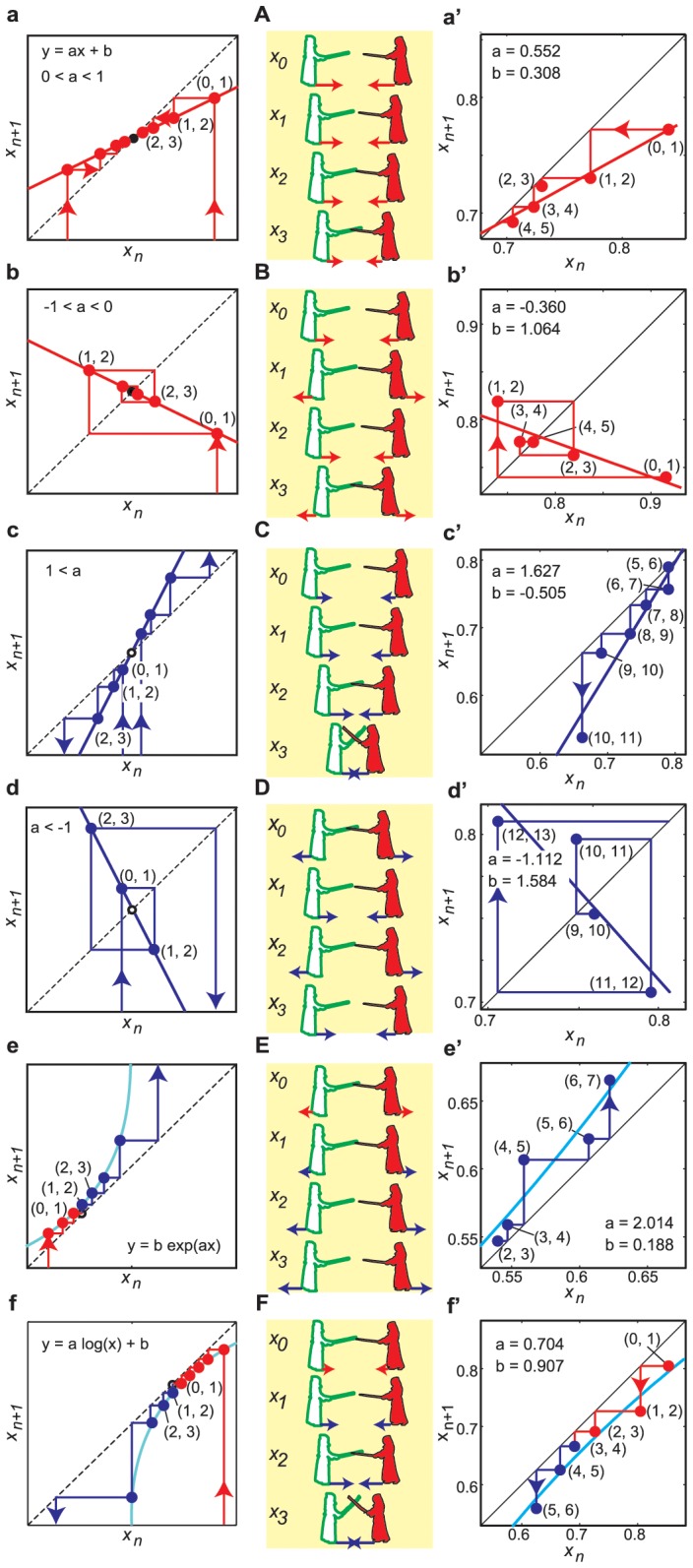
Trajectories of six functions and return map analysis. (**a–d**) Linear functions, 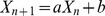, with four different slopes for 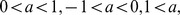 and 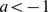, respectively. (**e**) Exponential function, 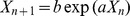. (**f**) Logarithmic function, 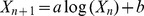. (**a**) Asymptotic trajectory to the attractive fixed point as a series of points, which corresponds to the movement of decreasing IPD by the step-towards motion shown in (**A**). (**a’**) Observed series of points in a scene from 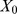 to 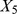, approaching an attractor with 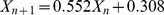. (**b**) Rotational trajectory to the attractor, which corresponds to the movement of decreasing IPD by alternating step-towards and step-away motions shown in (**B**). (**b’**) Observed series of points in a scene from 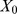 to 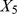, approaching an attractor with 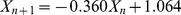. (**c**) Diverging from the repellent fixed point asymptotically, decreasing IPD by the step-towards motions shown in (**C**). (**c’**) Series of points (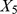 to 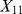), diverging from repeller with 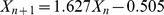. (**d**) Diverging from the repeller rotationally, increasing IPD by alternating step-towards and step-away motions shown in (**D**). (**d’**) Series of points (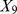 to 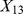), diverging from a repeller with 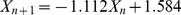. (**e**) Approaching and diverging trajectories around the attractor and/or the repeller exponentially, increasing IPD by step-away motions shown in (**E**). (**e’**) Series of points (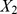 to 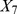), diverging from a repeller with 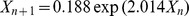. (**f**) Logarithmically approaching and diverging trajectories around an attractor, decreasing IPD by step-towards from motions shown in (**F**). (**f’**) Series of points (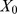 to 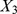) approaching an attractor and diverging from a repeller (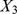 to 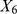) with 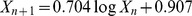.

The peak detection of 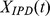 in each scene was calculated using a second-order Savitzky-Golay smoothing filter with nine points [35]. The mean intervals for scenes were 11.2 s 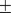 5.67 s for experts and 12.2 s±5.45 s for intermediate players. The peaks in each scene can be visualized using a plot, which is a type of a return map. Such a map plots the present peak 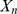 versus the next peak 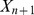. For each scene, we plotted the observed data as the present peak 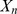 versus the next peak 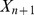 using the amplitude of 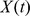 at the peaks of 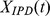 as a discrete dynamical system (Figure 2C–D), referred to as return map analysis. Periodicities are revealed on such a plot as intersections with the line of identity 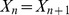 [36], [37]. These intersections are known as an attractive fixed point and repellers or saddle points. These attractive fixed points are deterministically approached from a direction called the stable direction or manifold, and the repellers are diverged from these attractive fixed points along the unstable direction or manifold as a linear function. Theoretically, we postulated the linear function, 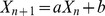. The intersections can be classified into two properties depending on the absolute value of 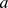. When 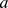 is less than 1, 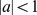, then the intersection is considered to be an attractive fixed point (i.e., an “attractor”). When the absolute value of 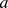 is more than 1, 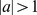, then the intersection is referred to as a repellent fixed point (i.e., a “repeller”). An attractor can be further classified into two types. When 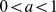 (Figure 3a), the trajectories asymptotically close to the attractor, that is, the IPDs decrease gradually (Figure 3A). When 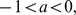 the trajectories rotationally close towards the attractor (Figure 3b); that is, the IPDs decrease by alternately moving a step towards and a step away from the attractor (Figure 3B). A repeller also has two types of trajectories: 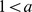, and 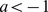, corresponding to asymptotical and rotational trajectories, respectively, as shown in Figure 3c, d and 3C, D. Trajectories also approach and diverge from points that do not cross the line 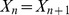. We postulated that these functions, an exponential function, 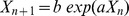 (Figure 3e, E), and a logarithmic function, 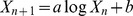 (Figure 3f, F), represent intermittency.

A total of 346 scenes with more than five peaks of 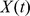 were fitted to three types of functions; 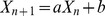, 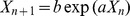, and 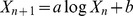. The number of fitted points was altered from three to six points on the return map using moving windows from the beginning of the data to the end of each scene:
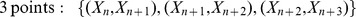


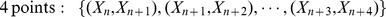


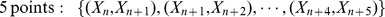


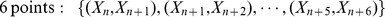



A plotted point on a return map corresponds to two consecutive peaks in the time series. Thus, the existence of 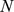 points for fitting means that 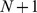 series of peaks in time series of IPDs were followed by certain regularities. As a measure of significance of fit, we used the 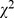 goodness-of-fit test and the incomplete gamma function, 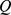. We excluded the results of a fit when the corresponding significance level exceeded 0.05; the minimum 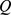-value was 0.824 for the remaining results; thus, these functions were identified with high confidence. If the same series of points were fitted by two different functions, then the series exhibiting lower 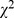 probability was selected. When the exponential and logarithmic functions were fitted to the series of points, the case in which the function was crossed 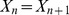 was excluded. Additionally, the longer series of points for the fitted function was selected, if the series was fitted by two different lengths of series.

Furthermore, to clarify the switching among several attractors and repellers, return maps were plotted using a well-fitted series of points as four different linear functions of an attractor and a repeller and histograms for each match were constructed from a well-fitted series of points according to the grouping of peak values in the bins. The threshold in each histogram, and the probabilities of second- and third-order state transitions were calculated for a well-fitted series of points as a linear function (Figure S3).

Calculations for function fitting, threshold determination, and transition probabilities were performed by programs written in the C-programming language, with several source files provided by “Numerical Recipes in C’’ [38].

## Results

### Return Map Analysis

**Figure 4:**
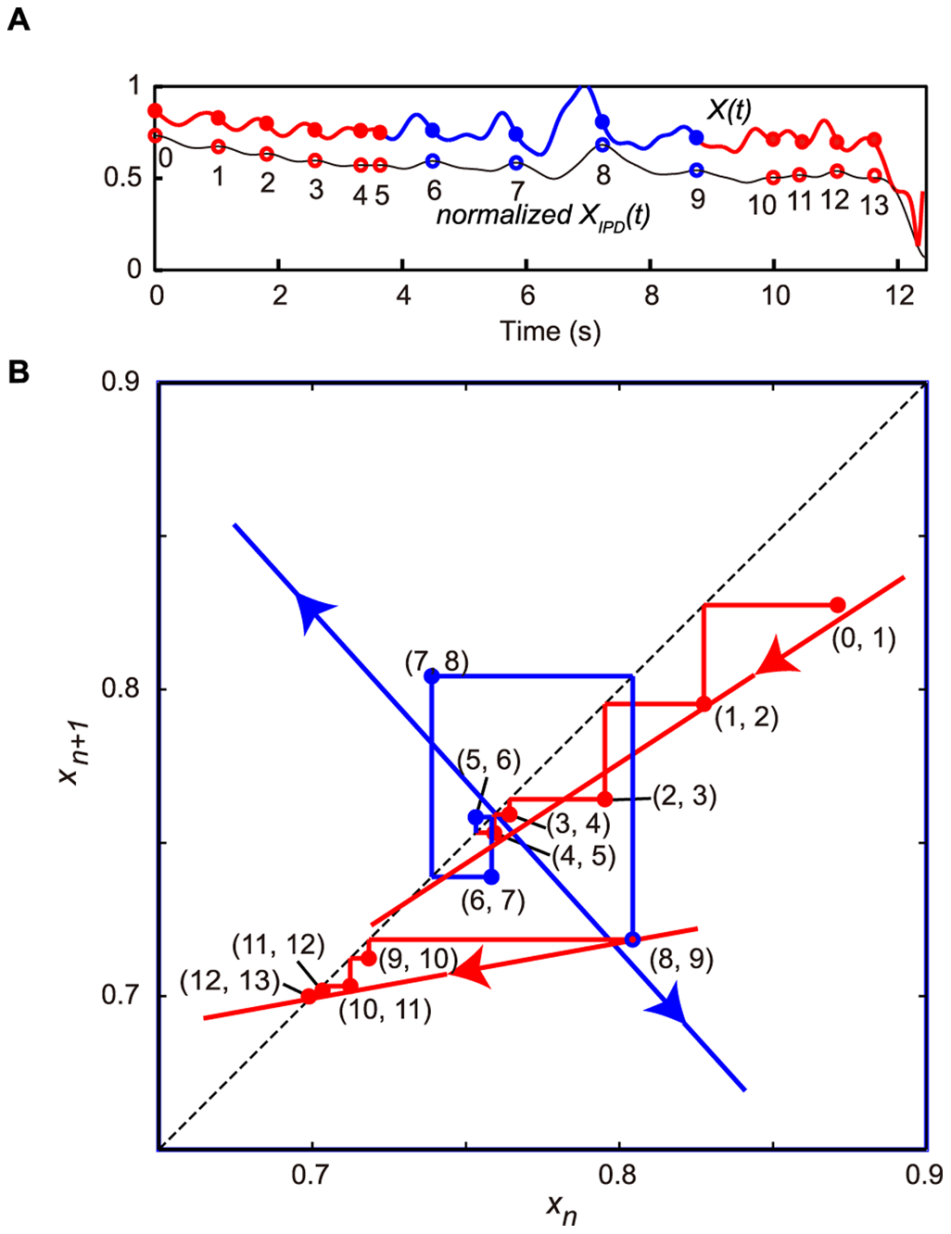
Switching among three different functions. (**A**) Time series of normalized 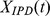 and state variable, 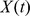, and circles show peaks of normalized 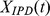. (**B**) Return map for this time series that demonstrates switching among three different functions. At first, from the peak of 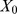 to 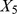, the observed series of points have approached to an attractive fixed point asymptotically with 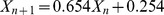. From the peak of 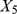 to 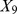, the observed points diverged from a repellent fixed point rotationally with 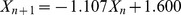. Finally, from the peak of 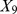 to 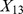, the observed points approached the other attractive fixed point asymptotically with 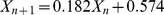.

**Figure 5:**
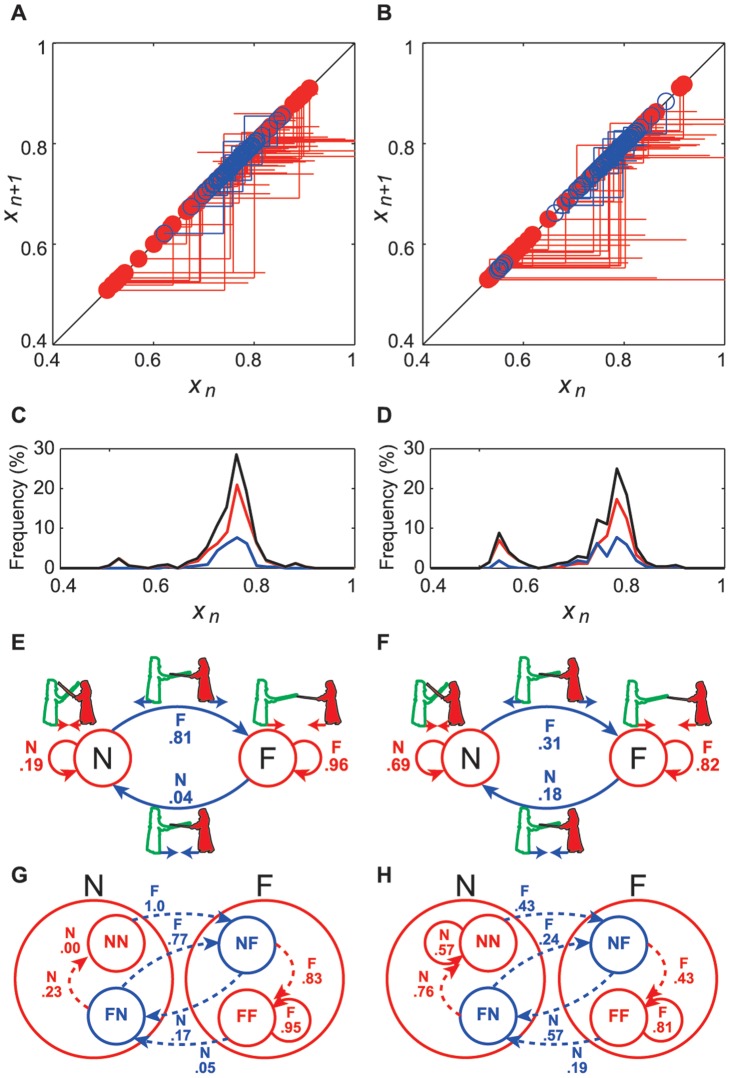
State transition diagrams. (**A, B**) Return maps were plotted using observed points as four different linear functions of an attractor (red) and a repeller (blue) for expert and intermediate competitors respectively. The circles show crossing points with the line of identity, 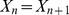. (C, D) Red, blue, and black lines show histograms of crossing points for an attractor, a repeller, and the sum of these respectively. (E, F) Second-order state transition diagrams with the conditional probabilities consisted of the “farthest apart” high velocity state (F) and the “nearest together” low velocity state (N) for expert and intermediate competitors, respectively. (G, H) The third-order state transition diagrams comprised four second-order sub-states.

For each scene, we plotted a return map of the time series of the observed data, 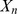 versus 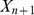, using the amplitude of 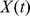 at the peaks of 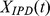. Figure 3a’–f’ shows examples of the fitting of six functions to the series of points on the return maps (Video S2 S4). We found 291 scenes that could be fit by the candidate functions: 162 of these scenes included expert competitors, and 129 scenes included intermediate competitors. In total, 485 series of points in these 291 scenes were well fit to the functions; 284 trajectories were revealed as attractors, and 146 trajectories were fitted as repellers; 55 trajectories were identified as intermittency (Table 1). All six types of candidate functions could be found using 3-, 4-, and 5-point fitting; 16 trajectories were fitted using 6 points for four types of functions. We found that 121 scenes were switched among two to nine different functions in each scene; 80 scenes switched between two functions, 22 scenes among three, 11 scenes among four, six scenes among five, and one scene each among seven and nine functions (Figure 4A–B, Table 2, Figure S4, and Video S5). These results suggest that complex movements occurring during the interpersonal competition of a kendo match could be generated by simple rules that attract toward or repel from fixed attractive and/or repellent points (Figure 5A–B).

**Table 1:** Numbers of well-fitted series of points by function fitting using three to six points from the series in a scene.

Function	Aa		Ar		Ra		Rr		Exp		Log		Sum	
Fitting points	E	I	E	I	E	I	E	I	E	I	E	I	E	I
3	61	35	42	47	17	23	39	28	6	7	8	5	173	145
4	33	19	4	5	12	14	3	1	1	2	10	9	63	50
5	17	11	2	–	–	4	1	–	–	1	2	–	22	16
6	5	3	–	–	1	3	–	–	1	–	2	1	9	7
	116	68	48	52	30	44	43	29	8	10	22	15	267	218
Sum	184		100		74		72		18		37		485	

Aa and Ar are asymptotical, 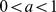, and rotational, 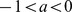, attractors. Ra and Rr are asymptotical, 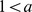, and rotational, 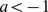, repellers. Exp and Log are intermittencies of exponential and logarithmic functions, respectively. E and I denote expert and intermediate competitors, respectively.

**Table 2:** Well fitted scenes switching between different functions in each scene.

Number of functions	1	2	3	4	5	6	7	8	9	Sum
Expert	100	39	14	4	3	–	1	–	1	162
Intermediate	70	41	8	7	3	–	–	–	–	129
Sum	170	80	22	11	6	–	1	–	1	291

The numbers show the different functions in each scene.

### State Transitions

We identified two discrete states in each histogram of return maps using the threshold as a minimum value for each match: the “farthest apart” high-velocity state (F), and the “nearest (closest) together” low-velocity state (N) (Figure 5A–D, and Figure S3). Thus, we identified four trajectories, 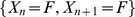, 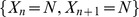, 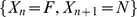 and 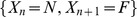, as second-order transitions. The state transition diagrams for experts and intermediate players are shown in Figure 5E and 5F, respectively. The conditional probabilities for second-order state transitions were calculated for each skill level. For experts, the transition probabilities of the four trajectories were: 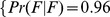, 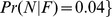, and 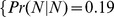, 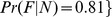, corresponding to two discrete states (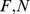). For intermediate players, the probabilities were:


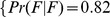
, 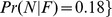, and 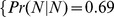, 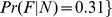. The differences in transition probabilities between experts and intermediates for each discrete state were significant according to Fisher’s exact test (F: 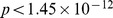, N: 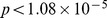). The offensive and defensive maneuvers of the experts were more often in the “farthest apart” high-velocity F-state. In contrast, those of intermediate players were more likely to be found in the “nearest (closest) together” low-velocity N-state. Two peaks can be observed in each histogram in the “farthest apart” high-velocity state (Figure 4C–D). This indicates that the current discrete state has two second-order states that depend on the previous state: 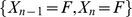 (FF) and 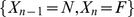 (NF). The probabilities of third-order trajectories between four second-order states were calculated for each skill level (Figure 4G–H) for experts and intermediates, respectively. For experts, the eight third-order transition probabilities were: 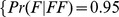, 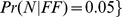, 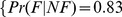, 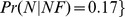, 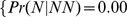, 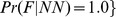, and 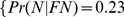, 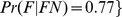, corresponding to four discrete states. For the intermediate players, the probabilities were:


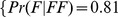
, 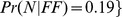, 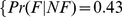, 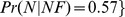, 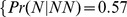, 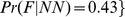, and 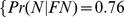, 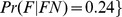. Experts exhibited higher probabilities for the third-order transitions to the state of “farthest apart” high velocity in all sub-states. On the other hand, intermediate players showed higher transition probabilities to the state of “nearest together” low velocity in all sub-states (FF: 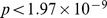, NF: 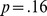, NN: 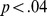, FN: 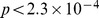). These results reveal that the second-order trajectories between two discrete states, that is, “farthest apart” high velocity and “nearest together” low velocity, and also the third-order trajectories among four discrete states, depend not only on the current state but also on the previous state. This suggests that these state transitions of offensive and defensive maneuvers in kendo have a hierarchical structure.

## Discussion

In this study, the return map analysis revealed that continuous interpersonal competition, which may appear to be quite complex, could be expressed in terms of a number of discrete dynamics represented by simple linear functions. The state transition analysis revealed second-order transition probabilities between two states: the “farthest apart” high-velocity state (F) and “the nearest (close) together” low-velocity state (N). These two states have a hierarchical structure that depends on the previous state. Third-order transition probabilities also revealed differences between expert and intermediate competitors. This result suggests that intentional switching dynamics is embedded in complex continuous interpersonal competition (such as a martial arts competition) and is thus better described as a hybrid dynamical system consisting of higher discrete and lower continuous modules connected via a feedback loop [28], [29]. This switching dynamic allows for very complex, diverse, continuous human movements.

Switching dynamics have been studied theoretically [39], numerically [40], and behaviorally [41] as a continuous dynamical system excited by a temporal input. This model accounts for the dynamics of switching among some attractors as fractal transitions within finite time intervals, as expressed in the following ordinary differential equation:
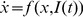
(6)where 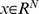 is the state of the system, and 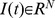 is the temporal input. This model has been extended to a hybrid dynamical system composed of a higher module with discrete dynamics and a lower module with continuous dynamics [28], [29]. The higher module selects the switching input 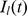 at the interval 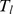 based on the following:
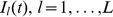
(7)where 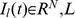, and 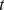 correspond to the 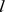
th input for the lower module, the number of inputs, and the time, respectively. The lower module can be described as a set of continuous non-autonomous dynamical systems [39], defined by the following ordinary differential equation:
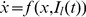
(8)where 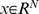 is the state of the lower module. The two modules are connected by a feedback system, in which the higher module switches at regular intervals in response to the states of the lower module. This system converges into various switching attractors that correspond to infinite switching manifolds; this defines the feedback control rule at the switching point. The feedback system could be considered to be an automaton that generates various sequences from the fractal set by choosing the typical switching manifold [28], [29]. This hybrid dynamical system could be considered a macroscopic model in which the discrete module corresponds to the brain function as decision making, and the continuous module corresponds to the motor function as human movements. Thus, the loop from the discrete to the continuous module can be regarded as an efferent pathway, and that from the continuous to the discrete module as an afferent pathway.

This idea is similar to neural syntax and could facilitate progress in defining cell assemblies, identifying their neuronal organization, and revealing causal relationships between assembly organization and behavior [31]. In general, syntax (grammar) is a set of principles that govern the transformation and temporal progression of discrete elements (e.g., letters and musical notes) into ordered and hierarchical relations (e.g., words, phrases, sentences, chords, chord progressions, and keys) that allow for a congruous interpretation of the meaning of language and music by the brain. In addition to its contribution to language and music, the grouping or chunking of fundamentals by syntax allows for the generation of a virtually infinite number of combinations from a finite number of lexical elements using a minimal number of rules in sign, body, artificial and computer languages, and mathematical logic. “Action syntax” [30] has been examined as a Markov chain in grooming behavior in rodents [32]–[34]. However, this behavior can be regarded as a stereotypical action that was generated by a relatively simple feed-forward excitatory mechanism. In contrast, interpersonal competition does not exploit this mechanism, because the environment changes abruptly and unpredictably. In response to various situations, very large numbers of movements can be generated in large strongly recurrently connected systems equipped with appropriate syntax [31]. The hybrid dynamical system [28], [29] in this study can be considered as a valid model for “joint action syntax”, which can generate various movements in interpersonal competition, such as martial arts.

In kendo, experts competed at greater distances and with higher velocities compared with intermediate competitors (Figure 5C–D). A point (ippon) in a kendo competition is achieved when an accurate strike is made on the opponent with the uppermost third of the shinai; (i.e., the top 0.30–0.40 m of its total length, 1.20 m). The average time of a strike movement was 0.36±0.08 s for the experts and 0.38±0.09 s for the intermediate players from an average interpersonal distance of 2.37±0.18 m for the experts and 2.42±0.18 m for the intermediate players. A split-second offensive or defensive maneuver may decide the outcome of a match. Thus, contestants must carefully maintain and change their interpersonal distance to balance the gain/loss of offensive and defensive maneuvers. This critical interpersonal distance, which induces the step-towards and step-away switching movement, has been shown in real settings [42], [43]. Our results suggest that experts engage in offensive and defensive maneuvers at greater distances, whereas intermediate players prefer closer distances for these maneuvers. Both players have similar attractors and repellers in their own matches; however, they play different movements based on the different syntax of their skill level.

Over the past several decades, intensive research has been conducted on emergent behavior in complex systems. In biological systems, in particular, research on a variety of complex systems has been focused on intelligence and the very nature of life itself. However, the intentional switching dynamics in interpersonal competition characterized by quick decision making and rapidly executed actions remain poorly understood. Higher-level cognitive brain functions generate seemingly homogeneous spatiotemporal sequences of neural activity to produce meaningful neural words and sentences in response to diverse environments. We can postulate a hybrid dynamical system that simulates decision making and/or intelligence [13], [14], [31]. Additionally, human movements are self-organized with robust autonomy not only in individuals but also in populations [20]–[22], [25], [27]. Joint action syntax, derived from hybrid dynamical system, is common and essential in nature from the level of neuronal activity to that of the activities of daily living. Furthermore, this model can be used to incorporate intentionality and robust decision making in the movement of artificial systems.

## Supporting Information

Figure S1 **Experimental setting and interpersonal distance (IPD). A** Experimental setting. The black triangles correspond to cameras (a total of eight). **B** Reflective markers attached to the back of the player’s head, back of his waist, both ankles, right knee, and the top of the shinai to detect movement. **C** Top view captured by an optical motion capture system. The bar shows the IPD between two markers attached to the back of the player’s head.
(EPS)

Figure S2
**Variances of 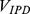 and correlation coefficients between 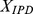 and 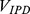 for each 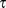.**
(TIF)

Figure S3 **Histograms of each match and its return map.** **A** and **C**, Examples of histograms of well-fitted peaks of each match. The thresholds were determined from the minimum frequency value of each match. The higher values of the peaks were regarded as “farthest apart” high-velocity states, denoted as “F”; the lower values of the peaks were regarded as “nearest (closest) together” low-velocity states, denoted as “N”. The threshold of **A** was 0.56, and the threshold of **C** was 0.6. **B** and **D**, Return maps corresponding to the histograms **A** and **C**, respectively, divided into four second-order sub-states: FF: Far-Far; FN: Far-Near; NN: Near-Near; and NF: Near-Far.
(TIF)

Figure S4 **Examples of return maps and switching function from a series of points in a scene.** Red lines show attractors, and blue lines show repellers. Cyan lines show intermittency in all panels. **A** Nine functions, **B** seven functions, and **C-H** five functions were switched sequentially.
(EPS)

Video S1 **Experimental setting and motion caputure data.** Animated clip showing an example of the experimental setting and an example of the motion capture data from side and top of views.
(MP4)

Video S2
**Attractor on the return maps.** Animated clip showing examples of the pattern of attractors on the return maps, which include the time series of 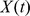 and 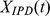, velocities of step-toward and step-away of each player, and 2D movement from top of view, and approaching to the attractive fixed point asymptotically and rotationally.(MOV)

Video S3
**Repeller on the return maps.** Animated clip showing examples of the pattern of repellers on the return maps, which include the time series of 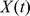 and 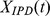, velocities of step-toward and step-away of each player, and 2D movement from top of view, and diverging from the repellent fixed point asymptotically and rotationally.(MOV)

Video S4
**Intermittency on the return maps.** Animated clip showing examples of the pattern of intermittencies on the return maps, which include the time series of 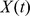 and 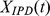, velocities of step-toward and step-away of each player, and 2D movement from top of view, and approaching and diverging around the attractive fixed point and/or repellent fixed point exponentially and logarithmically.(MOV)

Video S5
**Switching among several functions in one scene.** Animated clip showing examples of the pattern of switching among several functions on the return maps, which include the time series of 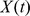 and 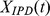, velocities of step-toward and step-away of each player, and 2D movement from top of view.(MOV)

## References

[1] Nicolis G, Prigogine I (1977) Self-organization in nonequilibrium systems : From dissipative structures to order through fluctuations. New York: Wiley.

[2] Haken H (1978) Synergetics: An introduction, non-equilibrium phase transitions and self-organization in physics, chemistry and biology. Berlin: Springer, 2nd edition.

[3] Lorenz EN (1963) Deterministic nonperiodic flow. J Atmos Sci 20: 130–141.

[4] Winfree AT (1967) Biological rhythms and the behavior of populations of coupled oscillators. J Theor Biol 16: 15–42. 6035757 10.1016/0022-5193(67)90051-3

[5] Kuramoto Y (1984) Chemical oscillations, waves, and turbulence. Berlin: Springer-Verlag.

[6] Strogatz S (2003) SYNC: The emerging science of spontaneous order. New York: Hyperion Books.

[7] Konishi S, Nakajima K, Uchida I, Kameyama M, Nakahara K, et al (1998) Transient activation of inferior prefrontal cortex during cognitive set shifting. Nature Neurosci 1: 80–84. 10195114 10.1038/283

[8] Miller EK (2000) The prefrontal cortex and cognitive control. Nature Rev Neurosci 1: 59–65. 11252769 10.1038/35036228

[9] Braver TS, Reynolds JR, Donaldson DI (2003) Neural mechanisms of transient and sustained cognitive control during task switching. Neuron 39: 713–726. 12925284 10.1016/s0896-6273(03)00466-5

[10] Hampton AN, Bossaerts P, O’Doherty JO (2006) The role of the ventromedial prefrontal cortex in abstract state-based inference during decision making in humans. J Neurosci 26: 8360–8367. 16899731 10.1523/JNEUROSCI.1010-06.2006 PMC6673813

[11] Huang XD, Ariki Y, Jack MA (1990) Hidden Markov models for speech recognition. Edinburgh: Edinburgh University Press.

[12] Erdös P, Rényi A (1960) On the evolution of random graphs. Publ Math Inst Hungarian Acad Sci 5: 17–61.

[13] Kozma R (2008) Intentional systems: Review of neurodynamics, modeling, and robotics implementation. Physics of Life Reviews 5: 1–21.

[14] Kozma R, Freeman WJ (2009) The KIV model of intentional dynamics and decision making. Neural Networks 22: 277–285. 19395236 10.1016/j.neunet.2009.03.019

[15] Haken H, Kelso JAS, Bunz H (1985) A theoretical model of phase transitions in human hand movements. Biol Cybern 51: 347–356. 3978150 10.1007/BF00336922

[16] Haken H (1983) Advanced synergetics: instability hierarchies of self-organizing systems and devices. Berlin: Springer.

[17] Haken H (2002) Brain dynamics: synchronization and activity patterns in pulse-coupled neural nets with delay and noise. Berlin: Springer.

[18] Kelso JAS, Scholz JP, Schöner G (1986) Nonequilibrium phase transitions in coordinated biological motion: Critical fluctuations. Phys Lett A 118: 279–284.

[19] Kelso JAS, Schöner G, Scholz JP, Haken H (1987) Phase-locked modes, phase transitions and component oscillators in biological motion. Physica Scripta 35: 79–87.

[20] Kelso JAS (1995) Dynamic patterns: The self-organization of brain and behavior. Cambridge, MA: The MIT Press.

[21] Schmidt RC, Carello C, Turvey MT (1990) Phase transitions and critical fluctuations in the visual coordination of rhythmic movements between people. J Exp Psychol: Hum Percept Perform 16: 227–247. 2142196 10.1037//0096-1523.16.2.227

[22] Ramenzoni VC, Davis TJ, Riley MA, Shockley K, Baker AA (2011) Joint action in a cooperative precision task: nested processes of intrapersonal and interpersonal coordination. Exp Brain Res 211: 447–457. 21479660 10.1007/s00221-011-2653-8

[23] Schmidt RC, Turvey MT (1994) Phase-entrainment dynamics of visually coupled rhythmic movements. Biol Cybern 70: 369–376. 8148414 10.1007/BF00200334

[24] Kugler PN, Turvey MT (1987) Information, natural law, and self-assembly of rhythmic movement. Hillsdale, NJ: Lawrence Erlbaum Associates.

[25] Frank TD, Richardson MJ (2010) On a test statistic for the Kuramoto order parameter of synchronization: An illustration for group synchronization during rocking chairs. Physica D 239: 2084–2092.

[26] Golubitsky M, Stewart I (2002) The symmetry perspective: from equilibrium to chaos in phase space and physical space. Basel, Switzerland: Birkhäuser Verlag.

[27] Yokoyama K, Yamamoto Y (2011) Three people can synchronize as coupled oscillators during sports activities. PLoS Comp Biol 7: e1002181. 10.1371/journal.pcbi.1002181 PMC3188505 21998570

[28] Nishikawa J, Gohara K (2008) Automata on fractal sets observed in hybrid dynamical systems. Int J Bifurcation Chaos 18: 3665–3678.

[29] Nishikawa J, Gohara K (2008) Anomaly of fractal dimensions observed in stochastically switched systems. Phys Rev E 77: 036210. 10.1103/PhysRevE.77.036210 18517488

[30] Lashley KS (1951) The problem of serial order in behavior. In: Jeffress LA, editor, Cerebral mechanisms in behavior: The Hixon Symposium, New York: Wiley. 112–136.

[31] Buzsáki G (2010) Neural syntax: cell assemblies, synapsembles, and readers. Neuron 68: 362–385. 21040841 10.1016/j.neuron.2010.09.023 PMC3005627

[32] Berridge KC, Fentress JC, Parr H (1987) Natural syntax rules control action sequence of rats. Behav Brain Res 23: 59–68. 3828046 10.1016/0166-4328(87)90242-7

[33] Berridge KC, Whishaw IQ (1992) Cortex, striatum and cerebellum: control of serial order in a grooming sequence. Exp Brain Res 90: 275–290. 1397142 10.1007/BF00227239

[34] Aldridge JW, Berridge KC (1998) Coding of serial order by neostriatal neurons: a ‘natural action’ approach to movement sequence. J Neurosci 18: 2777–2787. 9502834 10.1523/JNEUROSCI.18-07-02777.1998 PMC6793101

[35] Savitzky A, Golay MJE (1964) Smoothing and differentiation of data by simplified least squares procedures. Anal Chem 36: 1627–1639.

[36] Garfinkel A, Spano ML, Ditto WL, Weiss JN (1992) Controlling cardiac chaos. Science 257: 1230–1235. 1519060 10.1126/science.1519060

[37] Schiff SJ, Jerger K, Duong DH, Chang T, Spano ML, et al (1994) Controlling chaos in the brain. Nature 370: 615–620. 8065447 10.1038/370615a0

[38] Press WH, Teukolsky ST, Vetterling WT, Flannery BP (1992) Numerical Recipes in C: The art of scientific computing. Cambridge, England: Cambridge University Press.

[39] Gohara K, Okuyama A (1999) Dynamical systems excited by temporal inputs: Fractal transition between excited attractors. Fractals 7: 205–220.

[40] Gohara K, Okuyama A (1999) Fractal transition: Hierarchical structure and noise effect. Fractals 7: 313–326.

[41] Yamamoto Y, Gohara K (2000) Continuous hitting movements modeled from the perspective of dynamical systems with temporal input. Hum Mov Sci 19: 341–371.

[42] Kijima A, Kadota K, Yokoyama K, Okumura M, Suzuki H, et al (2012) Switching dynamics in an interpersonal competition brings about ‘deadlock’ synchronization of players. PLoS ONE 7: e47911. 23144834 10.1371/journal.pone.0047911 PMC3489899

[43] Okumura M, Kijima A, Kadota K, Yokoyama K, Suzuki H, et al (2012) A critical interpersonal distance switches between two coordination modes in kendo matches. PLoS ONE 7: e51877. 23284799 10.1371/journal.pone.0051877 PMC3527480

