# Supplementary figures and images for: Joint Action Syntax in Japanese Martial Arts
Source: PLoS One. 2013 Sep 4;8(9):e72436. doi: 10.1371/journal.pone.0072436 (PMC3762806; doi:10.1371/journal.pone.0072436)

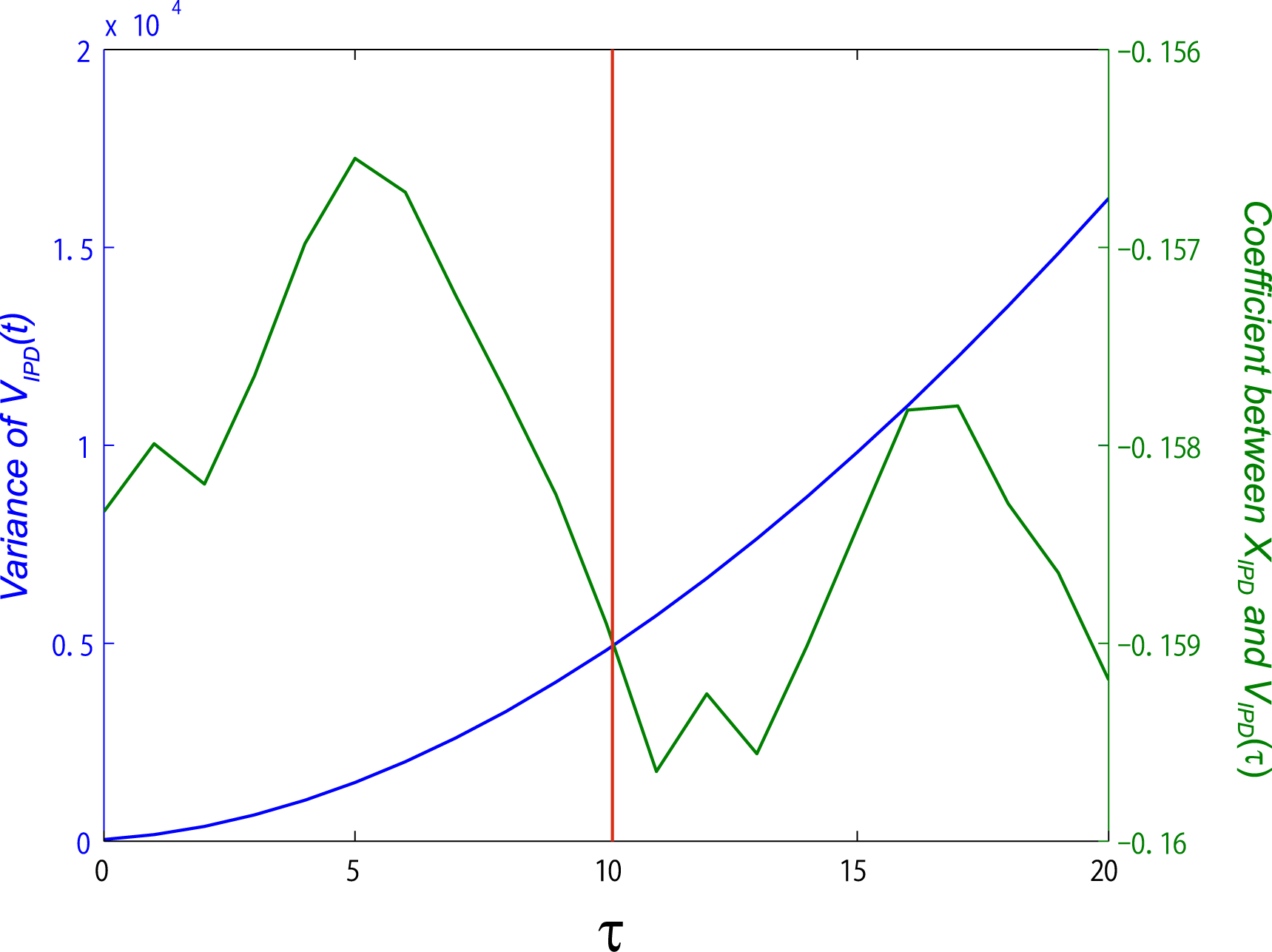

Supplement: Figure S2 — Variances of and correlation coefficients between and for each . (TIF) [file pone.0072436.s002.tif]

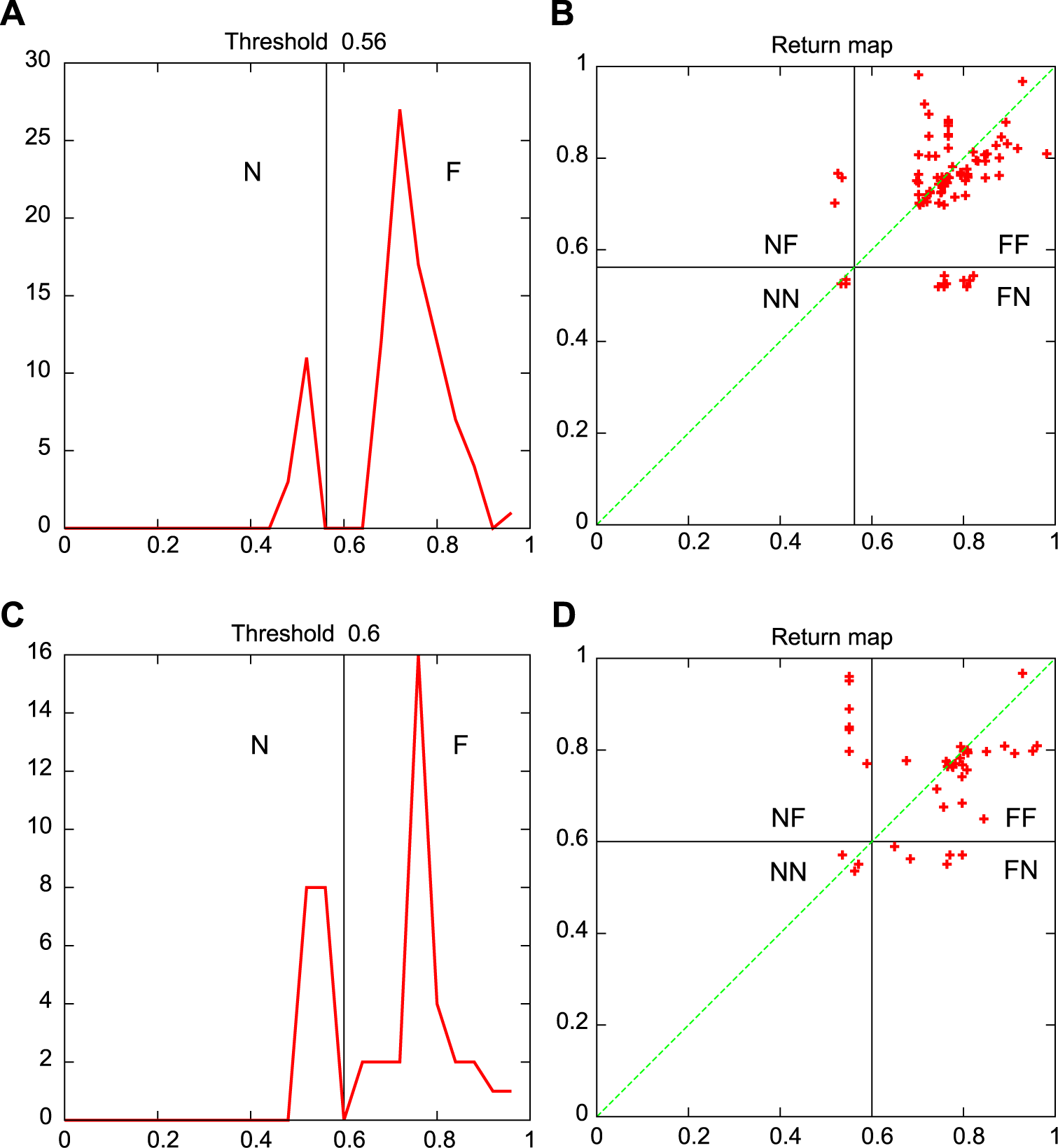

Supplement: Figure S3 — Histograms of each match and its return map. A and C, Examples of histograms of well-fitted peaks of each match. The thresholds were determined from the minimum frequency value of each match. The higher values of the peaks were regarded as “farthest apart” high-velocity states, denoted as “F”; the lower values of the peaks were regarded as “nearest (closest) together” low-velocity states, denoted as “N”. The threshold of A was 0.56, and the threshold of C was 0.6. B and D, Return maps corresponding to the histograms A and C, respectively, divided into four second-order sub-states: FF: Far-Far; FN: Far-Near; NN: Near-Near; and NF: Near-Far. (TIF) [file pone.0072436.s003.tif]
